# Analytical Complexity in Detection of Gene Variant-by-Environment Exposure Interactions in High-Throughput Genomic and Exposomic Research

**DOI:** 10.1007/s40572-016-0080-5

**Published:** 2016-01-25

**Authors:** Chirag J. Patel

**Affiliations:** Department of Biomedical Informatics, Harvard Medical School, 10 Shattuck St., Boston, MA 02215 USA

**Keywords:** Exposome, Genome, Genome-wide association study, Environment-wide association study, Gene-by-environment interaction

## Abstract

It seems intuitive that disease risk is influenced by the interaction between inherited genetic variants and environmental exposure factors; however, we have few documented interactions between variants and exposures. Advances in technology may enable the simultaneous measurement (i.e., on the same individuals in an epidemiological study) of millions of genome variants with thousands of environmental “exposome” factors, significantly increasing the number of possible factor pairs available for testing for the presence of interactions. The burden of analytic complexity, or sheer number of genetic and exposure factors measured, poses a considerable challenge for discovery of interactions in population-scale data. Advances in analytic approaches, large sample sizes, less conservative methods to mitigate multiple testing, and strong biological priors will be required to prune the search space to find reproducible and robust gene-by-environment interactions in observational data.

## Introduction

It is hypothesized that a portion of complex disease risk is due to interaction between inherited genetic and non-inherited environmental factors. Briefly in epidemiological research, an *analytic* gene-environment interaction can be defined when the association with a health outcome (e.g., estimate of disease risk such as a relative risk or odds ratio) for a gene factor and an environmental exposure is different when considered both factors alone (“main effects” in a statistical model) as compared to jointly [1]. In other words, the presence of an interaction implies that the health outcome of interest is different for an exposure *and* a specific genetic variant (or, equivalently, genetic risk for disease is different for alternate levels of exposure). In this perspective, we consider analytic challenges in the identification of gene-environment interactions in the context of “big data” and “omic” biomedical research.

While it seems intuitive that gene-by-environment interactions (*G* × *E*) exist, there are few reproducible and documented examples of *G* × *E* derived from human population or epidemiological cohorts (e.g., [2•, 3]). At the same time, advances in genomic technology have enabled investigators to ascertain hundreds to millions of variables, such as genetic variants (locations along the genome where individuals differ) in association with phenotypes. For example, *genome*-*wide association studies* (GWASs) are a type of investigation that allows investigators to search millions of genetic variants, or genotypes, in association with a disease or disease phenotype in large populations (numbering in the 10s to 100 s of thousands of individuals), resulting in robust and reproducible genetic associations in disease risk (e.g., [4–6]). Further, simultaneous ascertainment of multiple environmental factors of the *exposome* [7–12] may make possible an analogous search for environmental exposures associated with disease through *environment*/*exposome*-*wide association studies* (EWASs) [13].

The central hypothesis of a *G* × *E* investigation includes that disease risk (measured for instance with an odds ratio) for at least one exposure of the exposome under interrogation is different for at least one genetic variant configuration (e.g., a genotype) of the genome. To scale up the search for reproducible genetic variant-by-environment exposure interactions may seem as simple as leveraging emerging technologies of the genome and exposome. Here, however, we claim that despite advances in phenomic, genomic, and exposomic measurement technologies, data-driven search for reproducible genetic variant-by-environment exposure interactions in population-based data streams faces a computational challenge: analytic complexity. In the following perspective, we describe the analytic complexity and potential strategies for trimming the search space of potential hypotheses to find robust interactions in observational datasets. We first describe GWAS and EWAS.

### Primer: What Are GWASs?

With the sequencing of the genome and projects that characterized common genetic variation such as the HapMap and 1000 Genomes projects, investigators are now able to interrogate how genome-wide genetic differences in populations are associated with disease and disease-related phenotypes in epidemiological studies [14–16]. These revolutionary studies, known as “genome-wide association studies” (GWAS), have enabled investigators to ask what common genetic loci or single nucleotide polymorphism variants (SNPs) are associated with a particular phenotype in an agnostic, systematic, and comprehensive way with explicit control of multiple test correction to mitigate possibilities of false positive reporting. As of this writing, over 2000 GWAS investigating over 1500 traits have been documented in the NHGRI/EBI GWAS catalog [17] (also see: https://www.ebi.ac.uk/gwas/docs/downloads). The NHGRI/EBI GWAS catalog is a catalog of individual findings (or “summary statistics”) from over 2000 GWAS, such as odds ratios/*p* values of association, population ancestry of cohort, variant identifiers, and phenotype (e.g., disease or trait).

Specifically, during the HapMap and now 1000 Genomes projects, common single nucleotide (SNP) variants were cataloged on the basis of their population frequency (≥10 % population frequency), and major and minor allele versions [5]. The location of each SNP along the genome is referred to as a “locus” and the presence of variation at a particular locus denotes a “polymorphism” or a “polymorphic” locus. “Common” polymorphisms are those that occur at approximately greater than 5–10 % in the population. Thus, by definition, a “common” SNP must reside at a polymorphic locus. There are greater than 1 million common SNPs in the genome [15]. While SNPs are the most common type of polymorphism in the genome accounting for 90 % of genetic variation, many other types of genetic variation exist, such as copy number variants, insertions, and deletions.

GWAS associate traits to variants at common polymorphic loci in the genome and are enabled by genomic technologies, known as “SNP microarrays,” which can assay greater than 1 million loci simultaneously for an individual. These microarrays are now mere commodity items, making accessible genome-wide measurements on a large number of individuals [18]. Further, these technology platforms are known to have very low measurement error [19]. GWAS are constructed by recruiting thousands of individuals with (“cases”) and without (“controls”) a trait or disease (or health outcome). Genotype frequencies at each locus across the genome are then compared between cases and controls using common statistical tests such as chi-squared test [6], assuming independence between each locus.

### What is the Exposome and what is an EWAS?

The central promise of a unified way to measure the human *exposome* includes the discovery of novel environmental factors associated with and potentially causative of disease. The human exposome has been tentatively defined as the totality of environmental exposures such as dietary nutrients, pharmaceutical drugs, infectious agents, and pollutants encountered from birth to death [7, 8, 10, 12, 20•].

“Environment-wide association studies” or equivalently “exposome-wide association studies” (EWAS), which are analogous methodologically to GWAS, are a recently proposed analytic approach to systematically associate exposures with disease or disease-related phenotypes [13] and, e.g., [21, 22•, 23•, 24•, 25, 26•, 27•, 28]. In EWAS, multiple exposures are assessed simultaneously, but without considering interactions among them, for their association with a phenotype or disease of interest. The false discovery rate [29•] is controlled to adjust for multiple testing (discern signal from noise), and significant associations are validated in independent data (e.g., [23•, 24•, 27•]). The main advantage of this approach is that it systematically investigates an array of exposures and adjusts for multiple testing, thus avoiding selective reporting while enabling discovery. Just as the literature for genetic associations in disease has become more reproducible due to standardized and extensively validated analytical procedures [19], an analogous process to associate the exposome with disease and health outcomes may result in more robust environmental associations.

While EWAS is operationally similar to GWAS, many differences exist that pose challenges to the emerging paradigm. First, the environmental exposures are time-dependent—exposures to environmental agents and their biological effects vary considerably across the lifespan, from pre-conception (parental) exposure, in utero, through childhood into adulthood, and senescence. Second, exposures are spatially dependent and their biological effects can depend on the route of exposure. Third, the correlation structure of the exposome is dense—many environmental factors are correlated with many others [19, 30, 31•, 32•]—and it will be a challenge to identify the independent influence of a single exposure in disease. Another issue for causal inference includes confounding. Confounding refers to the phenomenon of an extraneous exposure being associated to the disease or trait of interest via the one actually being tested. GWAS contends with a few confounders, such as “population stratification” or race/ethnicity. Exposome-wide studies may be rife with confounding type of biases as indicated by dense correlational structure. While the exposome provides a promising way to potentially unify the measurement of many exposures, it is probably not realistic to expect continuous exposome monitoring of subjects. Instead, available data typically will comprise a few snapshots of exposure. Repeated longitudinal exposure data provide information about the longitudinal effects and can help avoid the problem of reverse causation. Furthermore, measurement error of environmental exposure measurements can significantly diminish chances to detect *G* × *E*. On the one hand, GWAS array measurement error is low (less than 1 % [33]); however, the error rates for individual exposure measurements can be much higher (for example, for mass spectrometry measurements for individual exposures may be greater than ten- to hundredfold higher, e.g., [34]). Measurement errors of *E* will stand in the way of detecting *G* × *E*.

Reverse causality, confounding, measurement error, and the time-dependence of environmental exposures are all issues that the analyst must deal with not only in exposome-wide research, but also in *G* × *E* interaction investigation. While these challenges are immense, we address here another type of analytic complexity: the large space of potential genetic variant by exposure interactions that are possible to test.

### Querying the Large Space of Possible Genetic Variant by Environmental Exposure Interactions

**Fig. 1:**
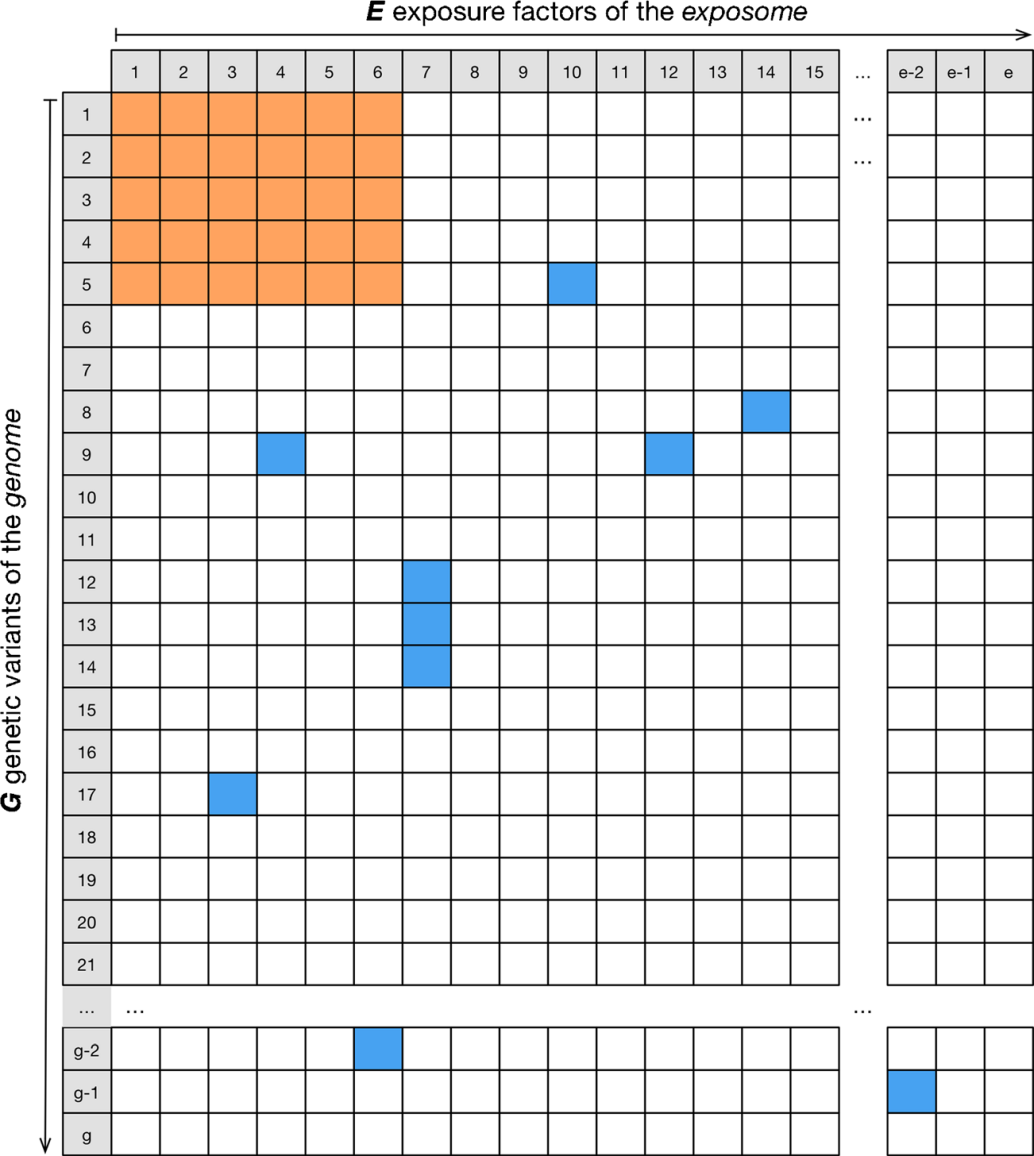
The “space” of all possible *G* × *E* interactions. Given *e* number of environmental exposures of the exposome and *g* number of genetic variants of the genome, each putative pair of exposome and genomic factors can be potentially tested for interactions in a disease; however, it may be a challenge to detect any interactions given the breadth of the space. Paring down the space of interactions to test (*seen in orange or blue*) will increase power for detection of interactions (Fig. 2)

In the following, we will refer to the potential *space* of genetic and environmental interactions that could occur as “analytic complexity” (Fig. 1). We will refer to the collection of genetic factors considered as *G* and environmental factors as *E*. Let us assume that genome-wide association study array technology and exposome-wide technology array can ascertain *g* number of genetic variants in *G* and *e* number of exposures in *E*, respectively. Therefore, the total space of possible interactions that could occur is *g* times *e* (Fig. 1). We claim that searching this large space of potential interactions is not tractable due to challenges in extracting signal from noise.

### Signal to Noise: Imbalance Between Type 1 Error and Power When Searching for Gene Variant by Exposome Factor Interactions

Generally, testing for a single pair of interactions is a power intensive task relative to testing a single genetic or environmental factor in association with disease risk. Inferring a gene-environment interaction requires testing for association between a single environmental exposure factor *E* in *each* stratum of the other single factor *G*. In contrast, detection of a *G* or *E* main effect without interaction requires no further stratification. In *G* × *E* analyses, the sample must be represented in all of the strata of *E* and *G*; thus, to ensure all strata are represented, larger sample sizes or *power* are required.

Power requirements are further exacerbated by attempting to search a number of *G* and *E* interactions simultaneously in association with disease. We claim there is a “signal-to-noise” challenge resulting in an imbalance between mitigating the chance for spurious findings and the requirement for large sample sizes.

What is meant by “signal-to-noise”? In essence, given *g* times *e* potential pairs of interactions, the central task includes how to infer those pairs of *G* and *E* that are indicative of a significantly different and larger effect size in a disease or in a phenotype (compared to the additive main effects of *G* and *E*). The central issue is that a comprehensive search of gene by environment interactions requires a much larger number of hypotheses compared to GWAS or EWAS alone. Concretely, given *g* number of genetic variants or *e* environmental exposures requires *g* or *e* number of hypothesis tests in GWAS and EWAS, respectively; however, to screen the possible space of interactions would require many more tests, equal to *g* times *e* (as depicted in Fig. 1). This leads to an increased chance for spurious results due to chance alone (type 1 error).

Type 1 error refers to a false positive: reporting an association, in this case an interaction, when the interaction does not exist. For example, imagine conducting an association study between 100 *G* and 100 *E* factors in blood pressure (a total of 100 * 100 = 10,000 possible interactions). Also, imagine that an oracle has told us the true scenario: that none of the interactions are truly associated with blood pressure (and therefore no associations should emerge). At a *p* value threshold of 0.05 under the distribution of no correlation between the 10,000 possible interacting factor pairs and blood pressure, 500 interacting pairs will emerge as significant due to chance! These are known as false positives and emerge when conducting more than one test of association. Therefore, the more tests one conducts, the more the absolute number of findings the investigator must sift through at typical levels of significance. The *family*-*wise error rate* is the chance of making at least one or more false findings, or discoveries, among all hypotheses. For example, in our large space of possible *G* and *E* interactions, the family-wise error rate would be indicative of the chance of a making one false discovery among all *G* × *E* tests. Therefore, to achieve low *p* values, larger sample sizes are required.

A method to control the family-wise error rate among all *G* × *E* tests includes the *Bonferroni correction*. With the Bonferroni correction, the significance threshold is divided by the number of tests. Take again our example of testing 100 *G* and 100 *E* factors. A Bonferroni significance threshold to guard against type I error for 10,000 interaction tests and for a typical significance threshold of 0.05 would be 0.05/10,000 or 5 × 10^−6^. Therefore the threshold to detect “signals” (a significant finding) must be stringent due to a large potential false positive “noise.”

Coupled with testing a number of strata in individual factors of *G* and *E*, the large number of potential hypotheses explored means that achieving significance requires great statistical *power*. Recall that *p* values are a function of the inverse of the standard error of the estimate, and standard errors are inversely proportional to sample size. Furthermore, in addition to sample size and the analytic complexity (or the number of interaction tests), the potential interaction effect size, the main effect and prevalence of exposure, and the main effect and prevalence of genetic variants all also influence power. Lack of *power* causes *type 2 errors*. Where type 1 error is the chance of a false positive, type 2 error gives the chance of false negatives (not detecting a discovery or signal when it does in fact exist). Power is the chance that the analyst will find an association provided it exists. Usually, analysts require power to be greater than 80 % to ensure high probability of a non-spurious association.

**Fig. 2:**
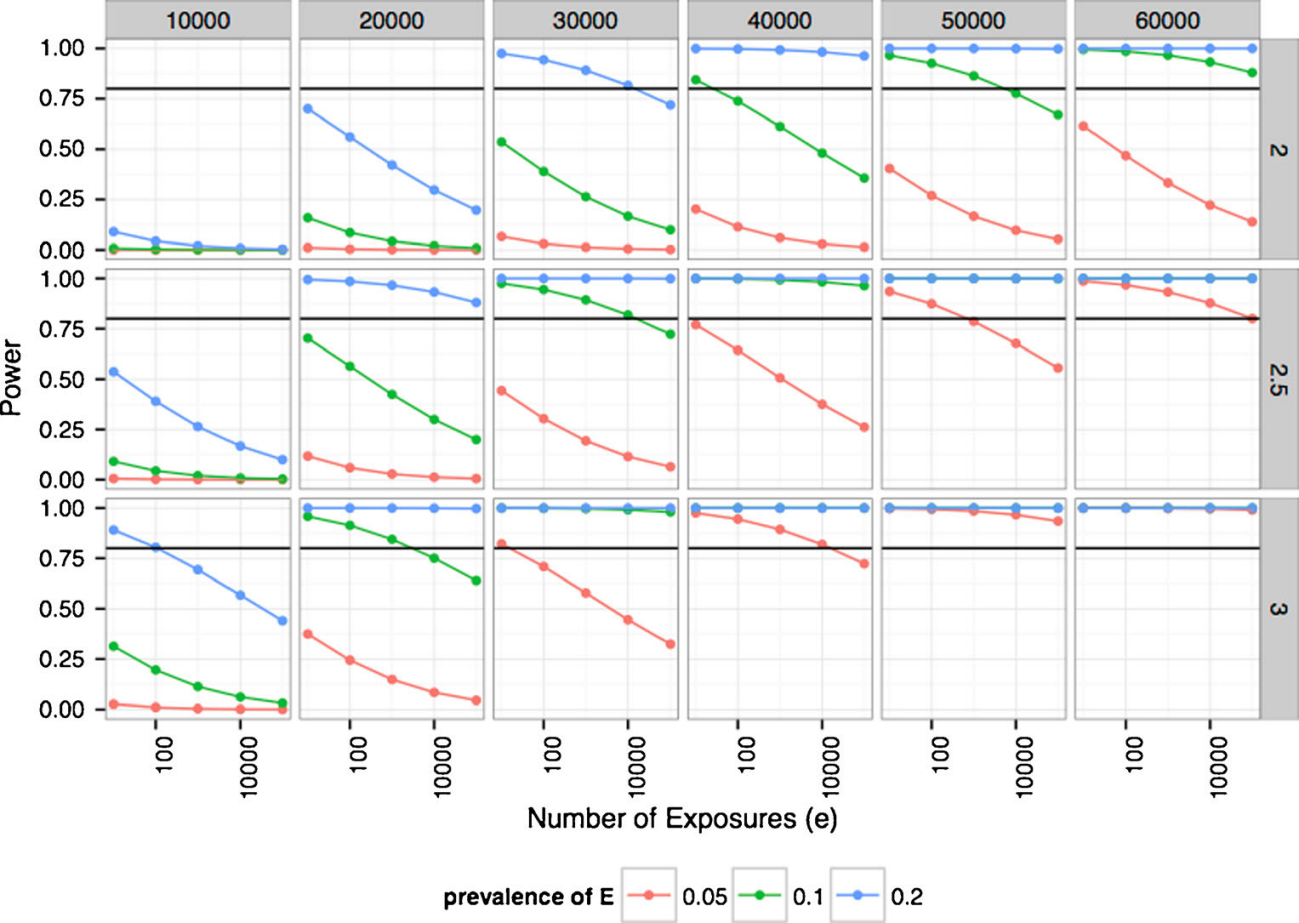
Power to search for one million SNPs by *e* number of environmental exposure interaction pairs as a function of number of factors of the exposome (*x*-*axis*), average exposure prevalence in the population (*red*: 5 %, *green*: 10 %, *blue*: 20 % prevalence), sample size (*in columns*), and effect size (odds ratio) for interaction (odds ratio for disease for both exposure and genetic variant versus neither) in rows. Other assumptions include disease prevalence is 10 % (e.g., type 2 diabetes prevalence), a case-control study (1:1 case:control ratio), the number of variants (*g*) is 1,000,000, risk variant frequency in the population is 10 %, and the main effects of each *G* is on average 1.1 (roughly what is observed in GWAS), and main effect of *E* is 1.5. *Black horizontal line* denotes 80 % power

We summarize the tension between type 1 and type 2 errors as a function of rising complexity in the number of interactions considered in the context of power (Fig. 2) for a typical case-control investigation (e.g., type 2 diabetics vs non-diabetics). Using the R package *powerGWASinteraction* [35], we estimated power for a case-control study for type 2 diabetes (assuming 10 % prevalence). We visualized power as a function of the number of exposures (*e*, which can include a number of binary factors, such as presence of a chemical exposure, infectious agent, dietary nutrients, or a sociodemographic attribute) potentially interrogated in the *exposome* (ranging from 100 to 100,000). We also visualize power as a function of interaction effect size, main effect odds ratio of genetic variant of 1.1 (as observed in GWAS e.g., [36]), main effect odds ratio of exposure of 1.5, prevalence of environmental exposure (e.g., 5, 10, 20 %), and total sample size of the case-control study. Critically, the power analysis assumes one million genetic variants in *G* are ascertained (common for a GWAS assay) and that the number of tests being performed is the number of exposures *e* multiplied by 1 million genetic variants. The Bonferroni significance threshold, a way to correct for the family-wise error rate as discussed above, is therefore 0.05/(*e* * 1 M SNPs).

The figure depicts that mitigating the analytic complexity for finding robust *G* × *E* will be a resource-intensive endeavor and require sample sizes not usually investigated in usual environmental epidemiology studies (greater than 10,000). First, the power for detecting interaction effect sizes in sample sizes of 10,000 are all low or below 80 % (left most column). In fact, detecting interactions between individual genetic and environmental exposure factors for sample sizes of 20,000 and interaction odds ratios of 2 is only possible for exposures that have prevalence greater than 10 % (see Fig. 2, plot for sample size of 20,000 and interaction odds ratio of 2.5, second plot from left and second from top). The most dramatic decrease in power occurs with exposures that have low prevalence (5 %, or red line). For comparison, recall that the US prevalence of smoking is roughly 16–20 % [37] and physical inactivity is roughly 15–20 % [38]. It may not be tractable to detect many *G* × *E* in the largest of current-day epidemiological investigations with less than 10,000 subjects. We will provide a web-based *G* × *E* power calculator here: http://chiragjpgroup.org/resources/ge_power/.

### Examples of Approaches to Search for *G* × *E*

What can be done to address the challenges of this vast search space and discriminating signal from noise and power deficiencies when searching for interactions between genome-wide genetic variants and exposome-wide environmental exposures? We propose “data-driven” methods that leverage existing biological knowledge and epidemiological findings can aid in paring down the “search” space (Fig. 1, orange or blue regions).

We claim the most straightforward approach includes *trimming* the search space by choosing candidate *G*’ variants (out of all possible *G*) and *E*’ exposure factors (out of *E* possible) to test. However, this task is also fraught with difficulty: what candidate factors does one choose? In current day gene-environment interaction studies, *G*’ variants and *E*’ factors are selected without sufficient documentation of the strength of their marginal associations (e.g., in a GWAS). One new way forward would be to screen a set of gene and environmental factors and use the strongest findings (larger relative effect sizes and replicated findings) from GWAS and EWAS, respectively, as candidates for further study and replication (Fig. 1, orange area). Specifically, the orange area (Fig. 1) depicts a pairwise search between *g*’ number of factors and *e*’ number of exposure factors that is smaller than testing the entire *g* times *e* space of variants and exposures. Therefore, the analytic complexity or the number of interaction pairs tested is smaller and therefore increases the opportunity for signal versus noise and type 1 error. However, this comes at a cost of not finding interactions with low and non-significant main effects.

To construct such a screen, we propose utilizing factors arising from comprehensive and systematic studies that have resulted in robust and replicated associations with disease of interest.

In fact, we have demonstrated such an approach in searching for interacting factors in T2D [39•]. Specifically, we selected 19 GWAS-implicated loci in T2D (replicated significant genetic main effects in T2D) (e.g., [36]). Second, we selected five EWAS-implicated environmental exposures in T2D EWAS [21]. These five environmental exposures were significantly associated with T2D in multiple cross-sectional cohorts. Therefore, we only examined a total of 19 factors in *G*’ and five exposures in *E*’ with strong main effects in GWAS and EWAS, respectively. This is equivalent to testing a total of 90 *G*’ *× E*’ pairs, a much smaller “space” than testing all genetic and environmental factors comprehensively. Further, we effectively diminished the required sample size to just under 2000 (albeit the discoveries require replication in other cohorts).

Bonferroni control of the family-wise error rate is also known to be conservative. In summary, Bonferroni correction guards against having at least one spurious finding; however, the potential cost is high requiring large sample sizes to achieve significance with increased possibilities of type 2 errors (low analytical power). Other more “powerful” methods that control for potential false positives such as the false discovery rate (FDR) [29•] exist to effectively raise the significance level for discovery. The FDR is the estimated proportion of false discoveries made versus the number of “real” discoveries made for a given significance level (e.g., 0.05). Briefly, rather than saying that we want to be 99 % sure that none of the discoveries are spurious, we state, using the FDR, a set of discoveries at a *p* value threshold that we think are drawn according to the null distribution (or “false discoveries”). This criterion allows this method to be a more powerful means to control for multiple testing. The most accessible approach for FDR correction is the Benjamini-Hochberg method, available in all major statistical analytics platforms and can be used to increase the power for *G* × *E*.

In the aftermath of over a thousand GWAS, investigators are now interested in how interactions may result in the detection of novel genetic variant associations. Discrimination of interaction effects in GWAS (also known as genome-wide interaction study (GWIS) [40•, 41]) is an active area of statistical research; however, most of these methods consider only a handful of discrete or binary environmental exposures at a time (in other words, genome-wide *G* by single *E*). Many of these methods are known as “two-step” approaches [42–47]. In the first step, a data-driven association scan is implemented to search for potential interactions. The second step is a formal testing of interactions on a limited subset of candidates generated in the first step. Some of these more powerful methods implement the first-step screen in only the cases (known as a “case-only” filter). A case-only filter simply tests the association between the genetic variant and exposure in only the cases. Interaction pairs that that are nominally significant in the “case-only” first step are passed on to the second step of interaction testing. The controls are considered in the second and formal step of interaction testing and the family-wise error rate is controlled for a much smaller subset of *G* by single *E* pairs and thereby increases power. Undoubtedly, advances have been made that preserve power gained in the case-only approach for screening but utilize controls (Li and Conti 2009; Mukherjee et al. 2012); however, the literature is scant with documented examples of genome-wide *G* by single *E* interactions uncovered through these methods. Much needs to be done to adapt these methods for higher throughput exposome-related research to tackle the sheer number of potential exposures measured in exposome research.

### Data-Driven Incorporation of Biological and/or Epidemiological Findings

While we discuss analytical complexity for uncovering potential *G* × *E*, we emphasize that statistical interaction does not mean biological interaction [40•]. But, on the other hand, we claim that utilization of emerging and rich biological sources of data can inform other potential signals from the large space of potential interactions (e.g., Fig. 1, blue potential interacting pairs).

For example, databases like the Comparative Toxicogenomics Database (CTD, [48, 49]) and the Toxic Exposome Database (TED, [50]) contain information and findings from documented toxicological or environmental health investigations. These databases can help narrow down the scope of *G* and *E* pairs interrogated. We describe an example using the CTD. As many readers will know, the CTD is a database containing manually curated data that describes how genes (e.g., transcripts, protein) respond to, or are influenced by, environmental toxicants in different organisms. As an example, one such toxicological relationship is derived from an investigation studying gestational exposure of the plasticizing agent bisphenol A on adipogenesis in a *Rattus norvegicus* model system [51], in which mRNA levels of the gene *LPL* increased after chemical exposure. In the CTD, 1.2 million toxicological toxicant-gene relationships published in diverse biomedical journals are curated. These 1.2 million relationships are summarized from CTD cover 11,547 unique toxicants and 39,000 genes from different species.

For example, let us suppose we want to undertake a *G* × *E* epidemiological investigation in T2D [52]. We can use the CTD to select genetic variants and environmental exposures to test in the investigation. In our documented example, we first take all the SNP loci discovered from T2D GWAS and locate the genes where the SNPs reside. We mapped 75 T2D GWAS loci (SNPs) with *p* values less than 1 × 10^−6^ to 35 genes. We then looked for toxicants/potential exposures that have relationships with these 35 genes in the CTD as well-reasoned documented factors to execute a *G* × *E* investigation. In this example, we selected genetic loci with strong prior main effects based on their GWAS associations and exposures from the CTD that putatively modulate gene function of GWAS implicated loci, such as gene expression, in effect selecting candidates with a priori biological and epidemiological evidence in the literature. We stress that interaction investigations should proceed to examine established environmental and genetic risk factors (e.g., [53•]). Extending these ideas, a general heuristic for testing a smaller space of interactions (e.g., Fig. 1 in orange or blue) to enhance reproducibility of findings may include the following:

1. Assess strength of association of genetic variants and environmental factors in multiple epidemiological studies, such as EWAS or GWAS, respectively, if available.
2. If EWASs are not available, assess strength of association of *E* in “field-wide” meta-analytic epidemiological studies in disease or phenotypic trait of interest, whereby a list of environmental factors are compiled based on a comprehensive list of current published studies [54] and, e.g., [55].
3. In parallel or in combination in steps 1 and 2, select *G* and *E* that have some evidence of biological interaction in databases such as CTD or T3DB.

## Conclusions

In summary, we claim that the burden of analytic complexity, or sheer number of *G* × *E* interaction tests made possible by emerging genomics and exposomics assays, poses a considerable challenge in the execution of data-driven searches for interactions in population-scale data. In the future, new analytic approaches, less conservative methods to mitigate multiple testing, and strong biological and/or epidemiological priors will be required to prune the search space to find reproducible and robust gene-by-environment interactions in observational data.
